# Cadmium-Induced Effects on Bone in a Population-Based Study of Women

**DOI:** 10.1289/ehp.8763

**Published:** 2006-02-02

**Authors:** Agneta Åkesson, Per Bjellerup, Thomas Lundh, Jonas Lidfeldt, Christina Nerbrand, Göran Samsioe, Staffan Skerfving, Marie Vahter

**Affiliations:** 1 Institute of Environmental Medicine, Karolinska Institutet, Stockholm, Sweden; 2 Department of Clinical Chemistry, Karolinska University Hospital, Huddinge, Sweden; 3 Department of Occupational and Environmental Medicine, University Hospital, Lund, Sweden; 4 Department of Community Health, Malmö University Hospital, Malmö, Sweden; 5 Department of Medicine and; 6 Department of Gynecology and Obstetrics, University Hospital, Lund, Sweden

**Keywords:** biochemical bone markers, bone mineral density, cadmium, lead, osteoporosis, women

## Abstract

High cadmium exposure is known to cause bone damage, but the association between low-level cadmium exposure and osteoporosis remains to be clarified. Using a population-based women’s health survey in southern Sweden [Women’s Health in the Lund Area (WHILA)] with no known historical cadmium contamination, we investigated cadmium-related effects on bone in 820 women (53–64 years of age). We measured cadmium in blood and urine and lead in blood, an array of markers of bone metabolism, and forearm bone mineral density (BMD). Associations were evaluated in multiple linear regression analysis including information on the possible confounders or effect modifiers: weight, menopausal status, use of hormone replacement therapy, age at menarche, alcohol consumption, smoking history, and physical activity. Median urinary cadmium was 0.52 μg/L adjusted to density (0.67 μg/g creatinine). After multivariate adjustment, BMD, parathyroid hormone, and urinary deoxypyridinoline (U-DPD) were adversely associated with concentrations of urinary cadmium (*p* < 0.05) in all subjects. These associations persisted in the group of never-smokers, which had the lowest cadmium exposure (mainly dietary). For U-DPD, there was a significant interaction between cadmium and menopause (*p* = 0.022). Our results suggest negative effects of low-level cadmium exposure on bone, possibly exerted via increased bone resorption, which seemed to be intensified after menopause. Based on the prevalence of osteoporosis and the low level of exposure, the observed effects, although slight, should be considered as early signals of potentially more adverse health effects.

Osteoporosis is characterized by low bone mass and microarchitectural deterioration of the skeleton, leading to fragility and increased risk of fractures (Consensus Development Conference 1993). Sweden is among the countries with the highest incidence of osteoporotic fractures (Ismail et al. 2002; Kanis et al. 2002), but established risk factors cannot fully explain the wide geographic differences and the increased incidence over time. Identification of risk factors is vital to prevent reduction of life quality and life expectancy and to minimize the high costs of treatment associated with the disease. Known predictors of low bone mass are older age, female sex, family history of osteoporosis, early menopause, physical inactivity, low body weight, low intake of calcium and vitamin D, smoking, and alcohol consumption (Genant et al. 1999; National Institutes of Health Consensus Development Panel on Osteoporosis Prevention, Diagnosis, and Therapy 2001).

The toxic effect of cadmium on bone became evident at the outbreak of Itai-itai disease in Japan, where severe renal and skeletal damage in women was associated with consumption of heavily cadmium-polluted rice (Kjellström 1992). Cadmium is a widespread environmental pollutant, present in food (mainly cereals, vegetables, and shellfish) and tobacco. It poses a threat to human health because of its long retention (decades) in the kidneys (Järup et al. 1998). Recent studies indicate that relatively low exposure may also affect the skeleton (Alfvén et al. 2004; Staessen et al. 1999), but the relationship is not well documented. Whether the effects are mediated directly on bone or are secondary to kidney damage is still unclear (Kjellström 1992).

In contrast to cadmium, lead accumulates in bone by the replacement of calcium, and the skeleton contains as much as 90% of the lead body burden (Berglund et al. 2000; Nilsson et al. 1991).

The aim of this study was to assess associations between cadmium retention and bone effects in women of upper middle age, the most susceptible part of the population, with regard to both cadmium retention (Nishijo et al. 2004) and osteoporosis (Ismail et al. 2002; Kanis et al. 2002). We examined indicators of bone status, reflecting both short-term effects [markers of bone metabolism and parathyroid hormone (PTH)] and long-term effects [bone mineral density (BMD)]. We addressed the question of whether the effects of cadmium are mediated through kidney damage. We also assessed the impact of bone remodeling on endogenous lead exposure.

## Materials and Methods

### Study population

The Women’s Health in the Lund Area (WHILA) study, a population-based study of all women 50–59 years of age in the community of Lund, in southern Sweden (*n* = 10,766), started in December 1995 (Lidfeldt et al. 2001). In June 1999, when 1,160 subjects remained to be examined, the study was extended to include health aspects of cadmium and lead (Åkesson et al. 2005). The participation rate was 71% (*n* = 820). The exclusion criteria were hypo- and hyper-parathyroidism (*n* = 4), rheumatoid arthritis (*n* = 7), or oral use of corticosteroid drugs (*n* = 6). We collected data on lifetime smoking, alcohol consumption, physical activity, and reproductive factors, including hormone replacement therapy (HRT), via a questionnaire. Body weight and height were measured. We obtained morning spot urine from 797 women and blood from 727 women. All samples were collected during 8 months from June 1999 through January 2000. The ethics committee at Lund University approved the WHILA study, and oral informed consent was obtained from each participant.

### Measurements

Exposure assessment was based on cadmium in blood as a measure of ongoing exposure (which we assume has been fairly constant over time) and cadmium in urine as a measure of body burden (cadmium in urine correlates well with cadmium in the kidney cortex; Järup et al. 1998; Orlowski et al. 1998). In addition, we measured lead in blood (indicator of exposure). All equipment used in the study was tested, and possible contamination was below the limit of detection (LOD). Blood cadmium [LOD = 0.12 μg/L; precision = coefficient of variation (CV) = 7.4%], blood lead (LOD = 0.26 μg/L; CV = 3.1%), urinary cadmium (LOD = 0.31 μg/L; CV = 8.5%), and urinary calcium (LOD = 1.6 mg/L; CV = 6.4%) were determined by inductively coupled plasma mass spectrometry (Barany et al. 1997). The analytical accuracy was good and is described in detail elsewhere (Åkesson et al. 2005).

We measured the following biochemical markers related to bone metabolism: PTH, osteocalcin, and bone alkaline phosphatase (bALP) in serum; and deoxypyridinoline (U-DPD) and calcium in urine. We used immunoassays to determine intact PTH (Elecsys; Roche, Mannheim, Germany), osteocalcin (ELSA-Osteo; CIS Bio International, Gif-Sur-Yvette Cedex, France), and U-DPD (Immulite 2000 Pyrilinks-D; DPC, Los Angeles, CA, USA) and immunoradiometric assay to detect bALP (Tandem-R Ostase; Beckman Coulter, Inc., Fullerton, CA, USA). The CVs were < 4% for PTH, 10% for osteocalcin, < 9% for U-DPD, and < 11% for bALP.

We measured BMD of the nondominant wrist (at the 8 mm distal position) using dual-energy X-ray absorptiometry (DXT 200; Osteometer MediTech, Inc., Hawthorne, CA, USA). We used a phantom for daily calibration of the instrument, and one technician performed all measurements. The measured BMD was automatically compared with a “reference” population furnished by the instrument supplier, giving *T*-scores, defined as (BMD*_o_* − BMD*_m_*)/SD, where BMD*_o_* is the obtained BMD, BMD*_m_* is the mean value for 20-year-old Danish female controls, and SD is the standard deviation in the same reference population. Osteopenia was defined as −2.5 < *T*-score < −1.0, and osteoporosis as *T*-score < −2.5, according to the World Health Organization (WHO), based on the measurement of proximal femur (WHO 1994).

The kidney-effect markers measured were estimated glomerular filtration rate (GFR), creatinine clearance, and human-complex–forming protein (protein HC), and *N*-acetyl-β-d-glucosaminidase (U-NAG) in urine, as described previously (Åkesson et al. 2005).

Because creatinine excretion is dependent on muscle mass, which in turn may predict BMD, we chose to adjust all urinary markers to the group mean urinary density (1.015 g/mL) instead of to urinary creatinine (Åkesson et al. 2005; Suwazono et al. 2005).

### Statistical analyses

Data from two independent groups of subjects were compared by the Mann-Whitney *U*-test. We used Spearman rank correlation (*r_s_*) or Kendall’s tau to assess univariate associations (*p* ≤ 0.1). In multiple linear regression models, each bone-related variable was evaluated in relation to cadmium, potential confounders (factors associated with both cadmium and bone) and effect modifiers (factors associated with bone). We explored possible interactions in the model. Because the season of sampling correlated with blood and urinary cadmium, BMD, PTH, U-DPD, and urinary calcium, it was included in the models. Residual and goodness-of-fit analyses indicated no deviation from a linear pattern in the regression models. The final regression model included, apart from cadmium, only statistically significant variables (*p* ≤ 0.05). All tests were two sided, and statistical evaluation was performed using SPSS (version 12.01; SPSS Inc., Chicago, IL, USA).

## Results

The main characteristics of the participants are shown in Table 1. Current smokers had, on average, cadmium concentrations three times as high in blood (1.1 vs. 0.30 μg/L) and 1.5 times as high in urine (0.76 vs. 0.45 μg/L) compared with never-smokers.

**Figure 1:**
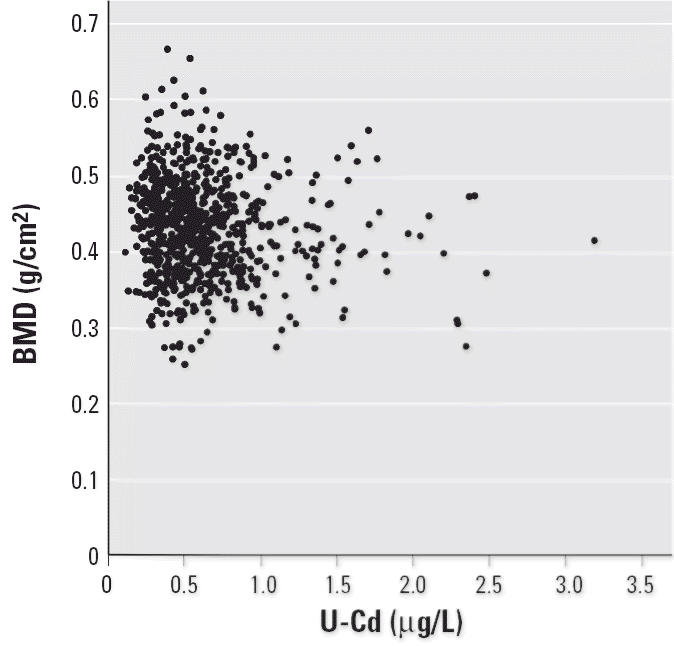
Association between forearm BMD and urinary cadmium (U-Cd) adjusted to density.

**Figure 2:**
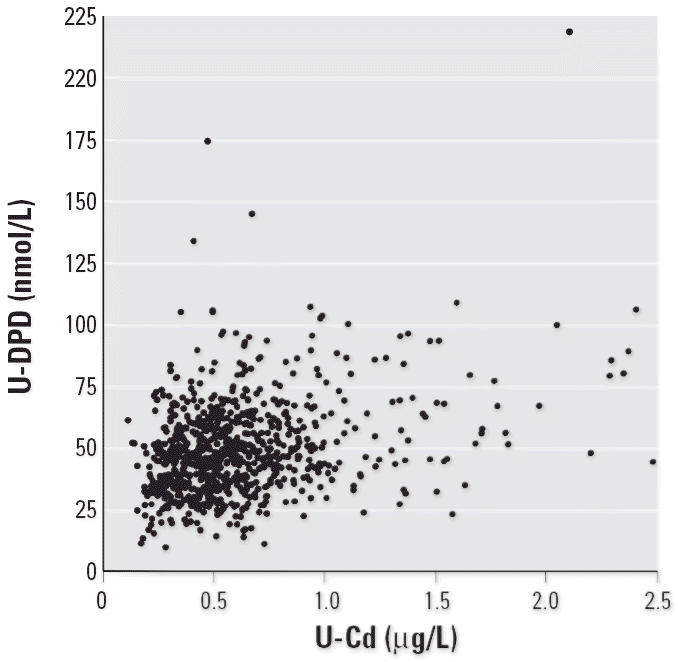
Association between U-DPD and urinary cadmium (U-Cd) adjusted to density.

In a first evaluation, we assessed the univariate associations between cadmium and the various bone-related variables and covariates (Table 2). Urinary cadmium was negatively associated with BMD (Table 2, Figure 1) and PTH, and positively associated with bALP and U-DPD (Table 2, Figure 2), but not with osteocalcin or urinary calcium. Similar but less pronounced associations were obtained for blood cadmium. Physical activity, parity, and total months of lactation were not associated with the bone-related variables.

Women on HRT had significantly higher BMD (*p* < 0.001) as well as lower osteocalcin, bALP, U-DPD, blood lead (*p* < 0.001), and urinary calcium (*p* < 0.023) than post-menopausal women without HRT. There were no differences in blood or urinary cadmium between the two groups.

### Multivariate analyses

In the multiple linear regression analyses, we evaluated further the associations between cadmium and bone by including potential confounders and effect modifiers (e.g., smoking and body weight, age, alcohol consumption, menopausal status including HRT, and age at menarche) (Table 3). We did not include blood lead as an explanatory variable in the models, in spite of the fact that it was associated with several of the skeletal biomarkers and BMD, because there is a known inverse relationship, that is, that skeletal demineralization releases lead into the blood. In the adjusted model, urinary cadmium, but not blood cadmium, showed a significant negative association (*p* = 0.047) with BMD. In a separate analysis in never-smokers with a lower cadmium exposure, the corresponding result was β = −0.02 g/cm^2^ per microgram per liter (*p* = 0.045; data not shown). Based on the adjusted model, we calculated the differences in BMD for the average woman with respect to age and weight at different levels of exposure. The exposure corresponding to the 99th percentile of urinary cadmium concentration had, on average, 5–6% lower BMD than those in the first percentile. This magnitude of difference was similar to that observed by a 6-year increase in age or an 11-kg lower body weight.

Both blood and urinary cadmium were negatively associated with PTH (Table 3); this was true for urinary cadmium even after excluding ever-smokers (β = −5.4 ng/L per microgram per liter; *p* = 0.027; data not shown). Urinary cadmium, but not blood cadmium, displayed a near-significant association (*p* = 0.06) with bALP. Further, urinary cadmium was associated with U-DPD (Table 3), even in never-smokers (β = 16 nmol/L per microgram per liter; *p* < 0.001; data not shown). The association between U-DPD and urinary cadmium was more pronounced (interaction term; *p* = 0.022) in post-menopausal women (β = 21 nmol/L per microgram per liter; Table 3) than in the HRT group together with premenopausal women (β = 12 nmol/L per microgram per liter).

In additional analyses, we evaluated the link between the cadmium-associated bone markers and the kidney effect markers. We included each kidney-effect marker in the multiple linear regression models for BMD, PTH, and U-DPD. Both urinary cadmium and U-NAG were associated with PTH and U-DPD but not with BMD. However, GFR and urinary protein HC were not associated with any of the cadmium-associated bone markers.

**Figure 3:**
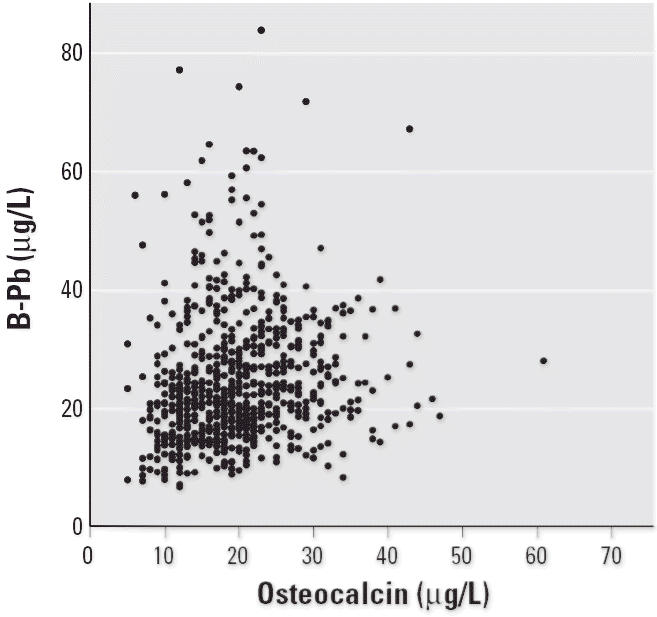
Association between blood lead (B-Pb) and serum osteocalcin.

Blood lead was higher in the post-menopausal women without HRT than in the HRT group and was associated with all bone parameters, except PTH (Table 2, Figure 3). Potential confounders were smoking and alcohol intake. Body weight and HRT were also associated with both blood lead and bone effects markers but are likely to be steps on a causal route rather than confounders. When body weight and alcohol were included in a multivariate model, significant associations with blood lead were still present for osteocalcin, bALP, U-DPD, and urinary calcium (all *p*-values ≤ 0.037). However, when HRT was included, the associations with blood lead became weaker (bALP and urinary calcium were no longer statistically significant, and the slopes decreased for osteocalcin and U-DPD).

## Discussion

This population-based study of upper-middle-age women, representative of the general population of southern Sweden, is the first to report assessment of a variety of biochemical bone markers in relation to cadmium exposure. We showed clear associations between increasing cadmium body burden, on one hand, and decreasing BMD, increasing bone resorption (U-DPD), and decreasing PTH, on the other. The associations persisted even in the group of never-smokers, which had the lowest cadmium exposure.

This study has several methodologic advantages, including the large sample size and high participation rate. Nevertheless, in health surveys, there may be a selection such that subjects with diseases, probably including those with bone disorders, participate to a lesser or to a greater extent than others. If fewer subjects with diseases participate, we may underestimate the risk of cadmium-induced bone damage; if more of these subjects participate, it may affect the generalizability of the results. However, the present prevalence of osteoporosis agrees well with the 7% value previously reported in Swedish women of the same age (Ringertz et al. 1995). Other advantages are the inclusion of several different markers of bone effects and confounders, and the fact that each individual’s exposure has been assessed separately, with good analytical accuracy. Any analytical imprecision would have caused a bias toward the null.

Urinary spot samples must be adjusted for dilution. This is frequently done by adjusting to urinary creatinine, but this method may induce bias because it is dependent upon muscle mass and thus affected by age and physical fitness (Suwazono et al. 2005), which are also predictors of bone status. In fact, creatinine-adjusted urinary cadmium displayed an even more pronounced statistically significant association with BMD. In view of this possible bias, we chose to correct by density (Åkesson et al. 2005; Suwazono et al. 2005). However, the choice is not obvious, especially not in a population as homogeneous for sex and age as that examined in the present study.

The cross-sectional study design precludes definite conclusions as to the direction of causality. The bone markers also showed clear associations with blood lead. Considering that about 90% of all the body burden of lead is localized to bone, even a minor increase of skeletal turnover, as in menopause, would affect the levels of lead in blood. Interestingly, the inclusion of HRT in the models decreased the strength of these associations, which indicates an effect of menopause and a protective effect of estrogen therapy, as previously shown (Garrido Latorre et al. 2003; Korrick et al. 2002; Nash et al. 2004; Vahter et al. 2004; Webber et al. 1995). Because the skeleton contains only minor amounts of cadmium (Petersson Grawe and Oskarsson 2000), it seems highly unlikely that an increased bone turnover would release significant amounts of cadmium.

This is the first study on cadmium-associated effects on bone in a population residing in an area with no known historical cadmium contamination, assuming a rather constant exposure over time. Nevertheless, our results are in accordance with findings of cadmium-associated effects on BMD and fractures in Swedes (of both sexes) with a similarly low present environmental exposure (Alfvén et al. 2000, 2004), Belgians with a somewhat higher exposure (Staessen et al. 1999), Japanese women (Honda et al. 2003), and Chinese men and women (Nordberg et al. 2002; Wang et al. 2003) with considerably higher exposure levels. In the present study, we obtained detailed information on several possible risk modifiers and confounders for osteoporosis, such as physical activity, menarche, menopausal status, and HRT. This enabled us to ascertain associations between low cadmium exposure and bone effects and our findings support a causal explanation. The effect of cadmium on bone resorption in our study was even more pronounced after menopause (interaction), in accordance with results from animal (Bhattacharyya et al. 1988) and human studies (Staessen et al. 1999), and in line with the fact that those affected by the Itai-itai disease were mainly women after menopause (Kjellström 1986).

Although the mechanism by which cadmium exerts effects on bone is far from clear, studies on humans have indicated an effect mediated through kidney damage (Alfvén et al. 2000; Horiguchi e al. 2005; Nordberg et al. 2002). We explored the mechanism by measuring markers of both bone metabolism and kidney effects. In contrast to previous reports, we suggest a direct effect of cadmium on bone resorption (osteoclasts), resulting in increased U-DPD. Such stimulation of bone resorption has been demonstrated in both animal and *in vitro* studies (Bhattacharyya et al. 1988; Brzoska and Moniuszko-Jakoniuk 2004, 2005; Carlsson and Lundholm 1996; Regunathan et al. 2003; Sacco-Gibson et al. 1992; Wilson et al. 1996). Because PTH is the main regulator of calcium metabolism, an increased bone resorption would lead to a compensatory decrease in PTH, which is in line with our results. The fact that we found no association between cadmium and bone formation (osteocalcin and bALP) may reflect a cadmium-induced uncoupling between bone resorption and formation (Uriu et al. 2000). In contrast, studies on patients with Itai-itai showed increased levels of markers of bone formation compared with controls (Aoshima et al. 2003; Kido et al. 1991; Tsuritani et al. 1994), and studies of other subjects with cadmium-induced tubular damage showed increased PTH (Nogawa et al. 1984; Tsuritani et al. 1992). This may indicate other mechanisms are involved in subjects with severe kidney damage.

An indirect effect on bone due to cadmium-induced kidney damage (Alfvén et al. 2000; Kjellström 1992), via impaired activation of vitamin D (Kjellström 1992; Nogawa et al. 1987), and increased excretion of calcium and decreased bone formation has been proposed. However, we found no association between cadmium and urinary calcium (Åkesson et al. 2005) or markers of bone formation (osteocalcin and bALP). This would indicate that the kidney was not involved, although the associations between the effect on bone and some of the renal effect markers may indicate some kidney-mediated effect.

Even though radius BMD is likely to reflect the risk of forearm fractures, it may not be a good index of osteoporosis in other parts of the skeleton, although there was correlation, albeit weak, between BMD of the radius and the hip in 81 women of the WHILA cohort (data not shown). Evaluation of cadmium exposure in relation to BMD of other sites associated with increased fracture risk, such as hip and lumbar spine, is required.

Clearly, the overall role of cadmium in the etiology of osteoporosis is limited. The observed difference in BMD between high-and low-exposed individuals corresponded to that of a 6-year increase in age or an 11-kg lower body weight. However, in view of the high prevalence of this disease, even a minor contribution is important at the population level. Furthermore, because the main cadmium exposure is via foods considered healthful and because everyone has lifelong exposure, our findings in combination with the observed effects on kidney (Åkesson et al. 2005) emphasize the importance of activities to reduce cadmium pollution of the environment.

## Figures and Tables

**Table 1:** Participant characteristics and data on exposure and bone-related variables in a population-based study of Swedish women.

	Median (5th–95th percentiles)	No. of samples
Population characteristics		
Age (years)	58 (54–63)	804
Weight (kg)	69 (54–94)	
Living alone or with children (%)	17	
Education > 12 years (%)	27	
Smokers: never/former/current (%)	55/24/22	
Pack-years: former/current smokers	10 (1–36)/20 (4–42)	
Alcohol consumption (grams ethanol/week)	20 (0–150)	
Menarche (age)	13 (11–16)	
Parity	2 (0–4)	
Lactation (months)	6 (0–24)	
Premenopausal/HRT/postmenopausal (%)	3/35/62	
Exposure variables		
Blood cadmium (μg/L)	0.38 (0.16–1.8)	715
Urinary cadmium (μg/L)^a^	0.52 (0.24–1.3)	795
Blood lead (μg/L)	22 (11–46)	716
Bone-related variables		
BMD (g/cm^2^)	0.44 (0.33–0.54)	803
Osteopenia, −2.5 < *T*-score < −1.0 (%)	42	
Osteoporosis, *T*-score < −2.5 (%)	7.2	
PTH (ng/L)	28 (13–57)	719
Osteocalcin (μg/L)	19 (9–33)	719
bALP (μg/L)	12 (6–21)	645
U-DPD (nmol/L)^a^	46 (25–85)	794
Urinary calcium (mg/L)^a^	135 (57–265)	797

Data are presented as median (5–95% percentiles) except as noted.

a Adjusted to mean density of 1.015 g/mL.

**Table 2:** Associations between exposure and bone-related variables (Spearman rank correlation coefficients).

	Blood cadmium	Urinary cadmium	Blood lead	Menarche	Pack-years of smoking	Alcohol	Weight	BMD	PTH	Osteocalcin	bALP	U-DPD	Urinary calcium
Age	−0.02	−0.03	−0.04	0.09*	−0.07†	−0.15**	0.06†	−0.18**	0.03	0.06	0.08*	−0.02	−0.04
Menarche	0.05	0.04	0.08*										
Pack-years of smoking	0.57**	0.41**	0.19**	0									
Alcohol	−0.01	0.02	0.36**	0.02	0.10*								
Weight	−0.11*	−0.15**	−0.07†	−0.12**	−0.07*	−0.08*							
BMD	−0.08*	−0.12**	−0.07†	−0.07*	−0.04	0.01	0.35**						
PTH	−0.10*	−0.17**	−0.06	NR	−0.10*	−0.12**	0.11*	0					
Osteocalcin	−0.03	0.05	0.23**	NR	−0.08*	−0.13**	−0.08*	−0.24**	0.17**				
bALP	0.02	0.08*	0.17**	NR	0.05	−0.09*	0.09*	−0.20**	0.16**	0.56**			
U-DPD	0.07†	0.27**	0.11*	NR	0.04	−0.07*	0.05	−0.05	−0.06	0.25**	0.23**		
Urinary calcium	−0.02	0.01	0.12*	NR	−0.01	0.03	−0.03	−0.12**	−0.11*	0.17**	0.16**	0.02	

NR, not relevant.

† 0.05 < *p* ≤ 0.10.

* 0.001 < *p* ≤ 0.05.

** *p* ≤ 0.001.

**Table 3:** Multiple linear regression models between bone-related markers and either urinary or blood cadmium.

	Urinary cadmium			Blood cadmium		
Dependent/independent variable	β	95% CI	*R*^2^	β	95% CI	*R*^2^
BMD (g/cm^2^)						
Cadmium (μg/L)^a^	−0.011	−0.022 to −0.0002	0.24	−0.002	−0.009 to 0.006	0.22
Weight (kg)	0.002	0.002 to 0.002		0.002	0.002 to 0.003	
Age (years)	−0.004	−0.006 to −0.003		−0.004	−0.006 to −0.003	
Menopause^b^		*p* < 0.001			*p* < 0.001	
Season^b^		*p* = 0.018			NS	
Menarche		NS			NS	
PTH (ng/L)						
Cadmium (μg/L)^a^	−4.3	−7.1 to −1.5	0.10	−2.2	−4.0 to −0.37	0.09
Weight (kg)	0.14	0.06 to 0.22		0.15	0.07 to 0.23	
Alcohol (g/week)	−0.03	−0.05 to −0.01		−0.03	−0.05 to −0.006	
Season^b^		*p* < 0.001			*p* < 0.001	
Pack-years		NS			NS	
bALP (μg/L)						
Cadmium (μg/L)^a^	0.95	−0.04 to 1.9	0.14			
Menopause^b^		*p* < 0.001				
Alcohol (g/week)		NS				
Weight (kg)		NS				
Age		NS				
U-DPD (nmol/L)						
Cadmium (μg/L)^a,^*	17	14 to 21	0.12	1.8	−0.7 to 4.4	0.03
Menopause^b^		*p* < 0.001			*p* < 0.001	
Season^b^		NS			NS	
Alcohol (g/week)		NS			NS	

Abbreviations: CI, confidence interval; NS, not significant; *R*^2^, explained adjusted variance for the total model.

a Adjusted to the mean urinary density.

b Three categories for menopause (HRT, premenopause, and postmenopause) and three categories for season (summer, fall, and winter) were included in the models as fixed factors (β not estimated).

* Significant interactions: urinary cadmium and menopause (*p* = 0.022).
